# Evaluating the Diagnostic Performance of Large Language Models on Complex Multimodal Medical Cases

**DOI:** 10.2196/53724

**Published:** 2024-05-13

**Authors:** Wan Hang Keith Chiu, Wei Sum Koel Ko, William Chi Shing Cho, Sin Yu Joanne Hui, Wing Chi Lawrence Chan, Michael D Kuo

**Affiliations:** 1 Department of Diagnostic and Interventional Radiology Queen Elizabeth Hospital Hong Kong China (Hong Kong); 2 Department of Clinical Oncology Queen Elizabeth Hospital Hong Kong China (Hong Kong); 3 School of Biomedical Sciences Li Ka Shing Faculty of Medicine The University of Hong Kong Hong Kong China (Hong Kong); 4 Department of Health Technology and Informatics The Hong Kong Polytechnic University Hong Kong China (Hong Kong); 5 Ensemble Group Scottsdale, AZ United States

**Keywords:** large language model, hospital, health center, Massachusetts, statistical analysis, chi-square, ANOVA, clinician, physician, performance, proficiency, disease etiology

## Abstract

Large language models showed interpretative reasoning in solving diagnostically challenging medical cases.

## Introduction

Large language models (LLMs) have demonstrated a surprising performance in radiological examinations [1]. However, their proficiency in real-world medical reasoning, especially when integrating multimodal data remains uncertain [2]. This study evaluates the ability of 3 commonly used LLMs—Google Bard (subsequently rebranded Gemini), Claude 2, and GPT-4—to generate differential diagnoses (ddx) from complex multimodality diagnostic cases.

## Methods

### Overview

**Figure 1:**
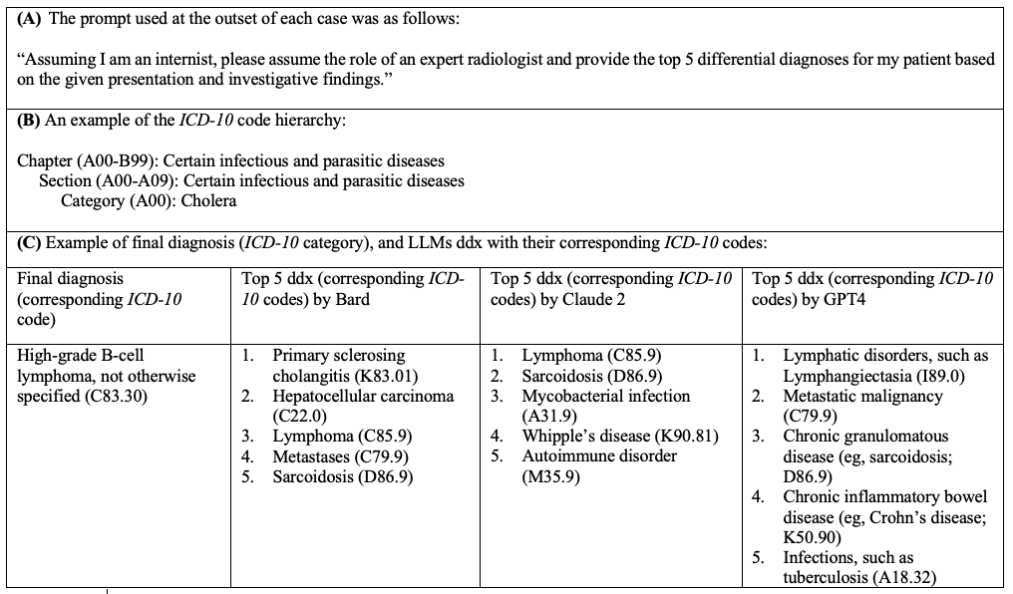
(A) Standardized prompt used for each case to generate differential diagnoses (ddx). (B) An example of *International Classification of Diseases, Tenth Revision* (*ICD-10*) code hierarchy structure; the first character (an alphabetical letter) denotes the chapter, and when combined with the next 2 digits, it forms the *ICD-10* category code. (C) An example of a large language model (LLM)–generated ddx and the corresponding *ICD-10* codes (case 34); in this case, none of the 3 LLMs included the final diagnosis (high-grade B-cell lymphoma, not otherwise specified; C83.30) in their ddx. For Bard, 3 of the 5 ddx belonged to the same chapter as the final diagnosis (chapter II: C22.0, C85.9, and C79.9). For Claude 2, only 1 of the 5 ddx belonged to the same chapter as the final diagnosis (chapter II: C85.9). For GPT-4, only 1 of the 5 ddx belonged to the same chapter as the final diagnosis (chapter II: C79.9).

Consecutive case records of the Massachusetts General Hospital from July 2020 to June 2023 were selected [3]. The cases were diagnostically challenging, but a final diagnosis was provided. Only the case presentation and a simple prompt asking for the top 5 ddx were used as input. Each case was run independently to prevent the model from being influenced by prior cases. To evaluate the stability of the results, all cases were reinputted into each LLM. To enable objective assessment, all diagnoses were mapped to their corresponding *International Classification of Diseases, Tenth Revision* (*ICD-10*) codes, with higher-level codes used in case an exact code could not be assigned (Figure 1).

The primary objective was accuracy, measured by whether the final diagnosis was within the LLM-generated ddx at the *ICD-10* category level. The secondary objectives were to measure the similarity between diagnoses within the ddx and the final diagnosis as well as their similarity to each other, measured at the *ICD-10* chapter level. Chi-square and ANOVA tests were used to compare categorical data between the LLMs. Statistical analyses were performed using Prism 10 (GraphPad Software).

### Ethics Approval

Approval from an institutional review board was not required due to the use of publicly available nonidentifiable data.

## Results

The diagnostic accuracy on 104 evaluated cases based on the first set of answers by the LLMs was 27.9% for Bard, 30.8% for Claude 2, and 31.7% for GPT-4. Accuracy significantly improved at the *ICD-10* chapter (body site or system) level, reaching 65.4% for Bard, 66.3% for Claude 2, and 71.2% for GPT-4. The mean number of the same ddx generated in each case in the repeatability testing was 2.3 (SD 1.1) for Bard, 2.4 (SD 1.2) for Claude 2, and 2.4 (SD 1.2) for GPT-4.

All 3 LLMs showed evidence of interpretive reasoning, as they tended to generate sets of ddx whose member diagnoses were often related to each other. The mean number of ddx per case belonging to the same *ICD-10* chapter as each other was 2.6 (SD 1.1) for Bard, 2.7 (SD 1.1) for Claude 2, and 2.4 (SD 0.9) for GPT-4. Interestingly, these related diagnosis “clusters” were often unrelated to the final diagnosis. The mean number of ddx belonging to the same *ICD-10* chapter as the final diagnosis was 1.2 (SD 1.3) for Bard, 1.4 (SD 1.4) for Claude 2, and 1.4 (SD 1.2) for GPT-4. These two findings were irrespective of whether the LLMs could include the final diagnosis in their ddx. Furthermore, the performance of the LLMs varied by disease etiology, although this difference was not statistically significant (Table 1).

**Table 1:** Performance of individual large language models (LLMs).

Characteristics		Bard	Claude 2	GPT4	*P* value	
**Accuracy by *ICD-10*^a^ hierarchy level, %**						
	Category	27.9	30.7	30.7	<.001^b^	
	Chapter	65.4	66.3	71.2	<.001^b^	
**Accuracy by *ICD-10* etiology (top 5 by frequency), n (%)**						
	Certain infectious and parasitic diseases (chapter I: A00-B99)	20 (35.0)	45.0	50.0	.62^c^	
	Neoplasm (chapter II C00-D48)	19 (52.6)	63.2	57.9	.75^c^	
	Diseases of the blood and blood-forming organs and certain disorders involving the immune mechanism (chapter III: D50-D89)	8 (12.5)	25.0	12.5	.74^c^	
	Endocrine, nutritional, and metabolic diseases (chapter IV: E00-E90)	9 (33.3)	33.3	33.3	>.99^c^	
	Diseases of the musculoskeletal system and connective tissue (chapter XIII: M00-M99)	11 (36.4)	72.7	63.6	.20^c^	
Number of diagnoses per ddx^d^ per case generated by LLMs belonging to the same hierarchical chapter as the final diagnosis based on assigned *ICD-10* codes, mean (SD)		1.2 (1.3)	1.4 (1.4)	1.4 (1.2)	—^e^	
Number of diagnoses per ddx per case generated by LLMs belonging to the same hierarchical chapter based on assigned *ICD-10* codes, mean (SD)		2.6 (1.1)	2.7 (1.1)	2.4 (0.9)	—	
Number of the same ddx per case generated by LLMs on repeatability testing, mean (SD)		2.3 (1.1)	2.4 (1.2)	2.4 (1.2)	—	

^a^*ICD-10*: *International Classification of Diseases, Tenth Revision*.

^b^Comparison of each LLM’s performance at the *ICD-10* category level versus the chapter level.

^c^Comparison of each LLM’s performance across different *ICD-10* etiologies. *P* values were not significant.

^d^ddx: differential diagnoses.

^e^Not applicable.

## Discussion

This study rigorously evaluated the diagnostic capacity of multiple LLMs using a simple standardized prompt [4]. The 3 LLMs represent state-of-the-art, general LLMs accessible to most clinicians. The relatively low accuracy of all 3 models at the *ICD-10* category level, coupled with a mean of >3 out of 5 diagnoses located in a chapter outside the final diagnosis chapter, collectively suggest either a knowledge or reasoning gap in current LLMs. Although performance differences are observed between different types of disease etiology (eg, 12.5% for Chapter III vs 63.6% for Chapter XIII in GPT4), the small numbers and unequal distribution of etiologies preclude adequate analysis; however, this area warrants further investigation. Conversely, the moderate number of LLM-generated ddx belonging to the same body site or system (chapter) implies these models can integrate and reason across complex clinical findings.

This study has limitations, including the low reproducibility of the ddx generated by the LLMs. The generative nature of these models and their continuous updates may lead to performance drifts and contradictory results. Further research and validation are necessary to generate consistent and explainable results as well as explore the relationships between performance and repeatability. Second, we did not assess whether human-artificial intelligence interaction or prompt engineering would affect diagnostic accuracy. Nevertheless, attempts to “overengineer” general LLMs toward a desired output could cloud real-world applicability, detracting from the ease of use that makes current LLMs attractive to general users [5]. Future work includes analyzing the rationales provided by the LLMs in reaching their ddx and asking the LLMs to quantify the likelihood of each ddx. Finally, the diversity of LLM-generated ddx warrants further exploration, as it could potentially hamper patient management [6].

In conclusion, LLMs may have a role in enhancing physician diagnosis of complex, multimodal clinical cases when applied judiciously.

## Abbreviations

Ddx: differential diagnoses
*ICD-10*: *International Classification of Diseases, Tenth Revision*
LLM: large language model
